# Celecoxib use and circulating oxylipins in a colon polyp prevention trial

**DOI:** 10.1371/journal.pone.0196398

**Published:** 2018-04-26

**Authors:** Jessica A. Martinez, Jun Yang, Betsy C. Wertheim, Denise J. Roe, Alexander Schriewer, Peter Lance, David S. Alberts, Bruce D. Hammock, Patricia A. Thompson

**Affiliations:** 1 University of Arizona Cancer Center, Tucson, Arizona, United States of America; 2 Department of Nutritional Sciences, University of Arizona, Tucson, Arizona, United States of America; 3 Department of Entomology, University of California Davis, Sacramento, California, United States of America; 4 UC Davis Comprehensive Cancer Center, University of California Davis, Sacramento, California, United States of America; 5 Department of Epidemiology and Biostatistics, Mel and Enid Zuckerman College of Public Health, University of Arizona, Tucson, United States of America; 6 Department of Pathology, Stony Brook University, Stony Brook, New York, United States of America; University Hospital Llandough, UNITED KINGDOM

## Abstract

Drugs that inhibit cyclooxygenase (COX)-2 and the metabolism of arachidonic acid (ARA) to prostaglandin E_2_ are potent anti-inflammatory agents used widely in the treatment of joint and muscle pain. Despite their benefits, daily use of these drugs has been associated with hypertension, cardiovascular and gastrointestinal toxicities. It is now recognized that ARA is metabolized to a number of bioactive oxygenated lipids (oxylipins) by cyclooxygenase (COX), lipoxygenase (LOX), and cytochrome P450 (CYP450) enzymes. Currently, the contribution of individual variability in ARA metabolism in response to the COX-2 inhibitors and potential adverse effects remains poorly understood. Using patient samples from the randomized, placebo-controlled phase III selenium/celecoxib (Sel/Cel) trial for the prevention of colorectal adenomatous polyps, we analyzed plasma concentrations of 74 oxylipins in a subset of participants who received celecoxib (n = 90) or placebo (n = 95). We assessed the effect of celecoxib (with and without low dose aspirin) on circulating oxylipins and systolic blood pressure (SBP). Individual CYP450- and LOX- but not COX-derived metabolites were higher with celecoxib than placebo (*P*<0.05) and differences were greater among non-aspirin users. LOX derived 5- and 8-HETE were elevated with celecoxib and positively associated with systolic blood pressure (*P* = 0.011 and *P* = 0.019 respectively). 20-HETE, a prohypertensive androgen-sensitive CYP450 metabolite was higher with celecoxib absent aspirin and was positively associated with SBP in men (*P* = 0.040) but not women. Independent of celecoxib or aspirin, LOX derived metabolites from ARA were strongly associated with SBP including 5- and 8-HETE. These findings support oxylipins, particularly the ARA LOX-derived, in blood pressure control and indicate that pharmacologic inhibition of COX-2 has effects on LOX and CYP450 ARA metabolism that contribute to hypertension in some patients.

## Introduction

Extensive work in animal and human studies led to the elucidation of prostaglandin E_2_ (PGE_2_), an oxidized lipid product of arachidonic acid metabolism via cyclooxygenase (COX), as a potent inflammatory molecule resulting in the development and widespread use of the NSAID as COX-1/2 enzyme inhibitors [1]. COX-inhibitors, including the selective and non-selective non-steroidal anti-inflammatory drugs (NSAIDs), are now among the most widely used drugs for their effectiveness in the treatment of fever, arthritis, and muscle and joint pain. Additionally, there is interest in their anti-cancer effects, particularly in the colorectum [2]. Despite the benefits, serious adverse effects of NSAIDs with chronic use, including heart failure that was initially identified in large cancer prevention trials [3, 4], continue to plague this class of drugs and challenge the safety of their use. For example, findings of adverse effects, particularly cardiovascular, led to the removal of rofecoxib (Vioxx), a potent selective inhibitor of COX-2, from the market and ultimately to black box warnings for all drugs in the NSAID class [5]. The FDA response was recently supported by findings from the PRECISION Trial demonstrating that the renovascular effects of COX-2 selective inhibitors (i.e., rofecoxib, celecoxib) are not limited to COX-2 selective NSAIDs. Indeed, celecoxib was found noninferior for cardiovascular toxicity to the commonly used, over-the-counter, non-selective NSAIDs ibuprofen and naproxen, and showed superior performance for gastrointestinal and renal toxicity to ibuprofen, but not naproxen [6]; these results confirm those of the Standard Care versus Celecoxib Outcome Trial [7] and meta-analysis of observational studies [8]. Importantly, these studies demonstrate the need to better understand the mechanism of renovascular effects of NSAIDs and individual susceptibility.

Less understood are the mechanisms that underlie the increased risk of hypertension, edema, and heart failure with chronic NSAID use in patients. NSAIDs, as a class, inhibit COX-mediated metabolism of arachidonic acid (ARA), a polyunsaturated fatty acid (PUFA). The PUFAs, including ω-3 [*e*.*g*., α-linolenic acid (ALA), eicosapentaenoic acid (EPA) and docosahexaenoic acid (DHA)] and the ω-6 fatty acids [*e*.*g*., arachidonic acid (ARA), linoleic acid (LA) and dihomo-γ-linolenic acid (DGLA)] are fatty acid precursor molecules for enzymatic oxidation to ‘oxylipin’ products. The oxylipins are a diverse group of lipophilic signaling molecules generated from PUFAs by the action of three major enzymes: cyclooxygenase (COX), lipoxygenase (LOX), and cytochrome P450 (CYP450) (S1 Fig). These bioactive lipids include the well-studied prostaglandins and thromboxanes as well as the mono-, di-, and tri-hydroxy fatty acids (FAs), epoxy FAs, lipoxins and other oxidized lipids that mediate an array of physiological effects including acting as regulatory molecules of inflammation [e.g., prostaglandin E_2_ (PGE_2_)] and as regulators of vascular tone and function [9]. This includes the oxylipin, prostacyclin I2 (PGI2), whose inhibition by the COX-2 inhibitors has been proposed as a mechanism of increased risk for the selective COX-2 inhibitors though their modest effects on the production of the potent platelet activator thromboxane (TX) A2. However, increases in hypertension and heart failure with the non-selective NSAIDs challenges this mechanism as the sole mediator of the cardiovascular effects of the NSAIDs [10].

**Fig 1:**
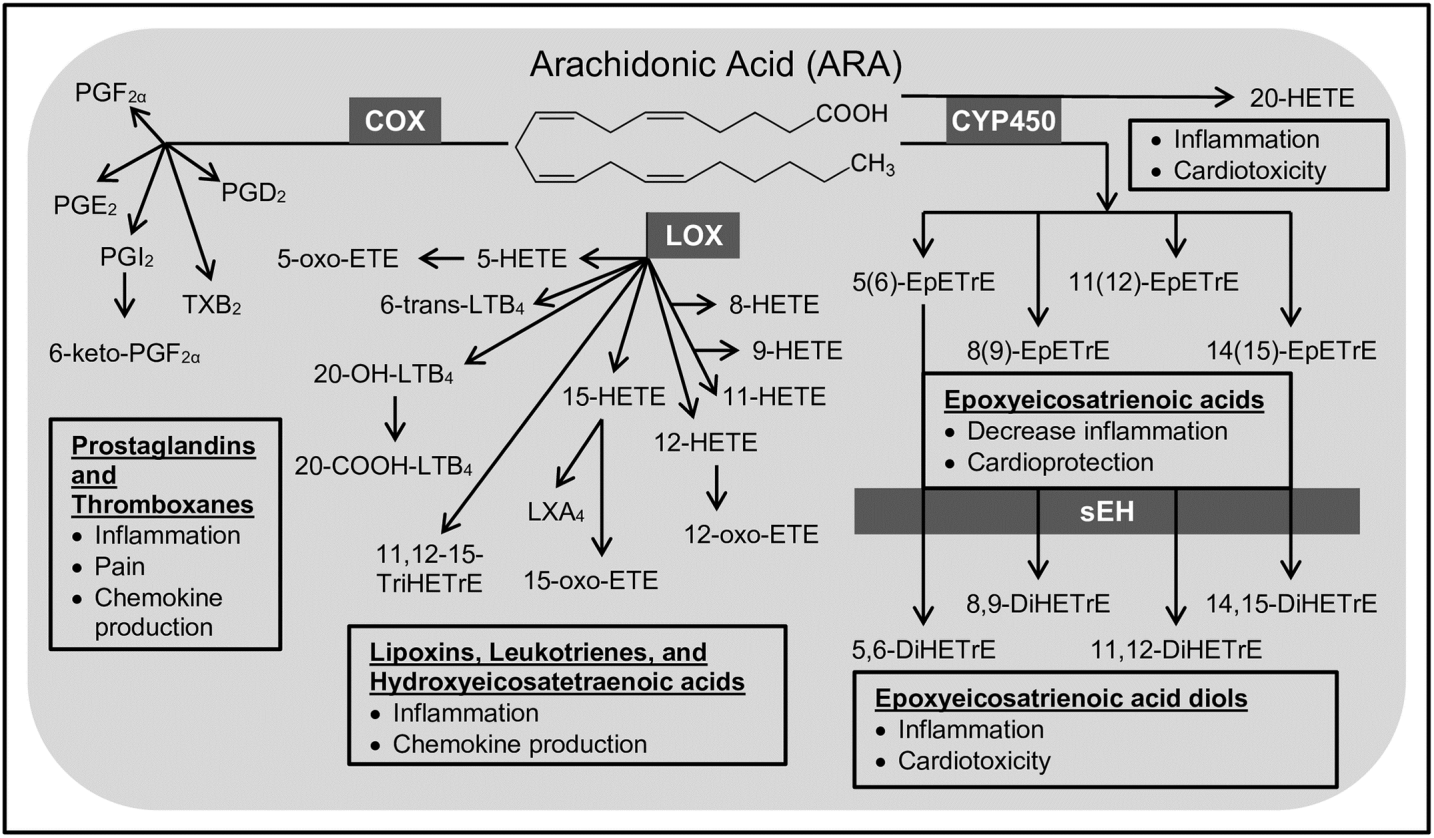
Arachidonic acid (ARA) pathway. ARA is metabolized by three enzyme families, cyclooxygenase (COX), lipoxygenase (LOX), and cytochrome P450 (CYP450), to produce numerous bioactive metabolites called oxylipins. Enzymes are shown in grey boxes. Oxylipin class and biological activity are outlined in black below the oxylipins they represent. The COX arm produces prostaglandins and thromboxanes that are pro-inflammatory, can cause pain, and can stimulate chemokine production. The LOX arm produces lipoxins, leukotrienes, and hydroxyeicosatetraenoic acids that are also pro-inflammatory and can stimulate chemokine production, with the exception of LXA_4_ which is anti-inflammatory. The CYP450 arm produces epoxyeicosatrienoic acids that decrease inflammation and are cardioprotective; however, these oxylipins are quickly metabolized by soluble epoxide hydrolase (sEH) to produce epoxyeicosatrienoic acid diols that are pro-inflammatory as well as cardiotoxic. The CYP450 arm also produces 20-hydroxyeicosatetraenoic acid (20-HETE), which is pro-inflammatory and cardiotoxic. Only oxylipins from the ARA pathway that were quantified on our analytical platform are depicted, excluding THF diol which is produced from multiple epoxyeicosatrienoic acids.

As a potent pro-inflammatory molecule, the ω-6 ARA-derived PGE_2_ produced from COX metabolism (Fig 1) is among the best-studied oxylipins as a drug target for its role in inflammation-associated diseases [11]. Importantly, while the COX-2 selective and non-selective NSAIDs (coxibs) were chosen for their inhibition of PGE_2_, it has subsequently been demonstrated that inhibition of PGE_2_ from ARA increases the generation of products of LOX- and CYP450-mediated metabolism of ARA, and select LOX- and CYP450-derived oxylipins produced from these enzymes effect vascular tone and blood pressure [12–15]. For instance, our group showed dramatically elevated levels of CYP450-derived 20-hydroxyeicosatetraenoic acid (20-HETE), a prohypertensive oxylipin, in male mice treated chronically with rofecoxib. Demonstration that 20-HETE also induced vascular effects of rofecoxib in treated mice led us to hypothesize that increases in 20-HETE may explain rofecoxib cardiovascular toxicities observed in patients [14].

Further supporting the adverse effects of COX-2 inhibition on blood pressure, we recently reported increased hypertension, particularly among men, in the Selenium and Celecoxib (Se/Cel) Phase III, placebo controlled, randomized study of 400 mg daily celecoxib for the prevention of colorectal adenoma [16]. Here we extend our investigation of COX-2 inhibitor effects on circulating plasma oxylipin concentrations and their relation to blood pressure in a subset of participants in the Sel/Cel Trial.

## Materials and methods

### Study population

The Selenium and Celecoxib Trial (Sel/Cel) was a randomized, double-blind, placebo-controlled phase III clinical trial of selenium (200 μg daily as selenized yeast), celecoxib (400 mg daily), the combination of selenium and celecoxib, or double placebo for the prevention of colorectal adenoma (the Sel/Cel Trial; ClinicalTrials.gov Identifier NCT00078897 [16–18]). In the parent trial, there were 824 participants that were randomly assigned to celecoxib or placebo. Concentrations of 74 oxylipins were obtained in the 12-month plasma sample for a subset of study subjects (*n* = 185). For the primary analysis of the sub-study presented here, only randomization to celecoxib or its placebo were considered (i.e. celecoxib alone and celecoxib plus selenium versus selenium alone and double placebo).

### Plasma sample collection and preparation

After collection, fasting plasma samples were immediately stored at -80°C and never thawed until oxylipin profiling. Plasma samples were prepared as previously described [19]. Briefly, once thawed, triphenylphosphine and butylated hydroxytoluene (0.2% w/w) were added to 250 μL plasma. The sample was then spiked with a set of deuterated isomers of 9 target analytes (including hydroxyeicosatetraenoic acids, thromboxanes, epoxides, prostaglandins, and diols) contained in 10 μL methanol and was then subjected to solid phase extraction. The collected eluents were evaporated to dryness using a centrifugal vacuum concentrator and re-constituted with 50 μL methanol solution with 1-cyclohexyl-dodecanoic acid urea as an internal standard. The spiked samples were vortexed and centrifuged before transfer to high performance liquid chromatography (HPLC) vials for analysis.

### Reverse phase chromatography with HPLC-MS

Oxylipin profiling was performed using an Agilent 1200 HPLC (Agilent, Santa Clara, CA) with AB Sciex 4000 QTRAP mass spectrometer (Sciex, Redwood City, CA). Acquisition parameters were as previously described [20] with minor modifications. The system was operated with scheduled MRM scan mode to overcome sensitivity loss with the increased number of analytes. Surrogate analytes and internal and external standards were used to monitor extraction efficiency and ensure accurate quantitation. The acquired data were quantified by Multiquant Software (Sciex, Redwood City, CA) using 9 isotope-labeled internal standards.

### Statistical analysis

Median oxylipin levels were compared across trial arms using non-parametric Wilcoxon rank-sum tests. Due to the known effects of aspirin on oxylipin levels, these tests were repeated after stratifying the study sample according to aspirin use at baseline. Further tests were conducted for aspirin (or NSAID) users versus non-users, in both trial arms combined. Oxylipin pathways were characterized by summing individual metabolites within ARA pathway arms, and these sums were compared across trial arms. Several analyses were stratified by sex, aspirin use, and/or NSAID use. Associations between oxylipins and systolic blood pressure (SBP) were tested using multivariate linear regression, adjusted for age, body mass index (BMI), aspirin use, hypertension medication use, and celecoxib assignment. Potential interactions between individual oxylipins and sex on SBP were tested using likelihood ratio tests. Statistical analyses were conducted using Stata 14.2 (StataCorp, College Station, TX). Given the limited sample size, high correlation between measures, and hypothesis-generating nature of the study, no adjustments were made for multiple comparisons. Oxylipin concentrations are presented in nM as median [interquartile range (IQR)].

## Results

### Patient characteristics

Placebo and celecoxib arms were balanced in terms of number of males, average age of the participants, and selenium randomization (Table 1). Trial arms were also balanced in terms of BMI, waist circumference, lipid profiles, diabetes and lipid-lowering medication use. There were more aspirin users, never-smokers, and hypertension-medication users in the placebo group. The celecoxib group had a slightly higher personal history of colorectal polyps and family history of colorectal cancer.

**Table 1:** Baseline characteristics of selected sample from the Sel/Cel Trial: Mean ± SD or *n* (%).

Characteristic	Placebo	Celecoxib
	(*n* = 95)	(*n* = 90)
Male	66 (69.5)	60 (66.7)
Age (y)	61.0 ± 9.4	63.1 ± 10.1
Cigarette smoking		
Never	44 (46.8)	34 (38.6)
Previous	41 (43.6)	49 (55.7)
Current	9 (9.57)	5 (5.68)
Personal history of colorectal polyps	23 (24.2)	27 (30.3)
History of colorectal cancer in a first-degree relative	24 (25.8)	24 (28.2)
Body mass index (kg/m^2^)	29.4 ± 5.2	28.8 ± 4.8
Waist circumference (in)	40.2 ± 6.4	39.9 ± 5.9
Total cholesterol (mg/dL)	198 ± 30	200 ± 35
High-density lipoprotein (mg/dL)	48.8 ± 14	52.0 ± 15
Triglycerides (mg/dL)	141 ± 72	153 ± 115
Diabetes	7 (7.37)	6 (6.67)
Aspirin, regular use	46 (48.4)	36 (40.0)
Hypertension medication	41 (43.2)	34 (37.8)
Lipid-lowering medication	32 (33.7)	29 (32.2)
Selenium randomization	47 (50.5)	46 (49.5)

Missing data: waist (*n* = 4), smoking (*n* = 3), history of polyps (*n* = 1), family history of colorectal cancer (*n* = 7).

### Effect of COX-2 inhibition on COX-, LOX-, and CYP450-derived metabolites from ω-6 or ω-3 fatty acids

**Fig 2:**
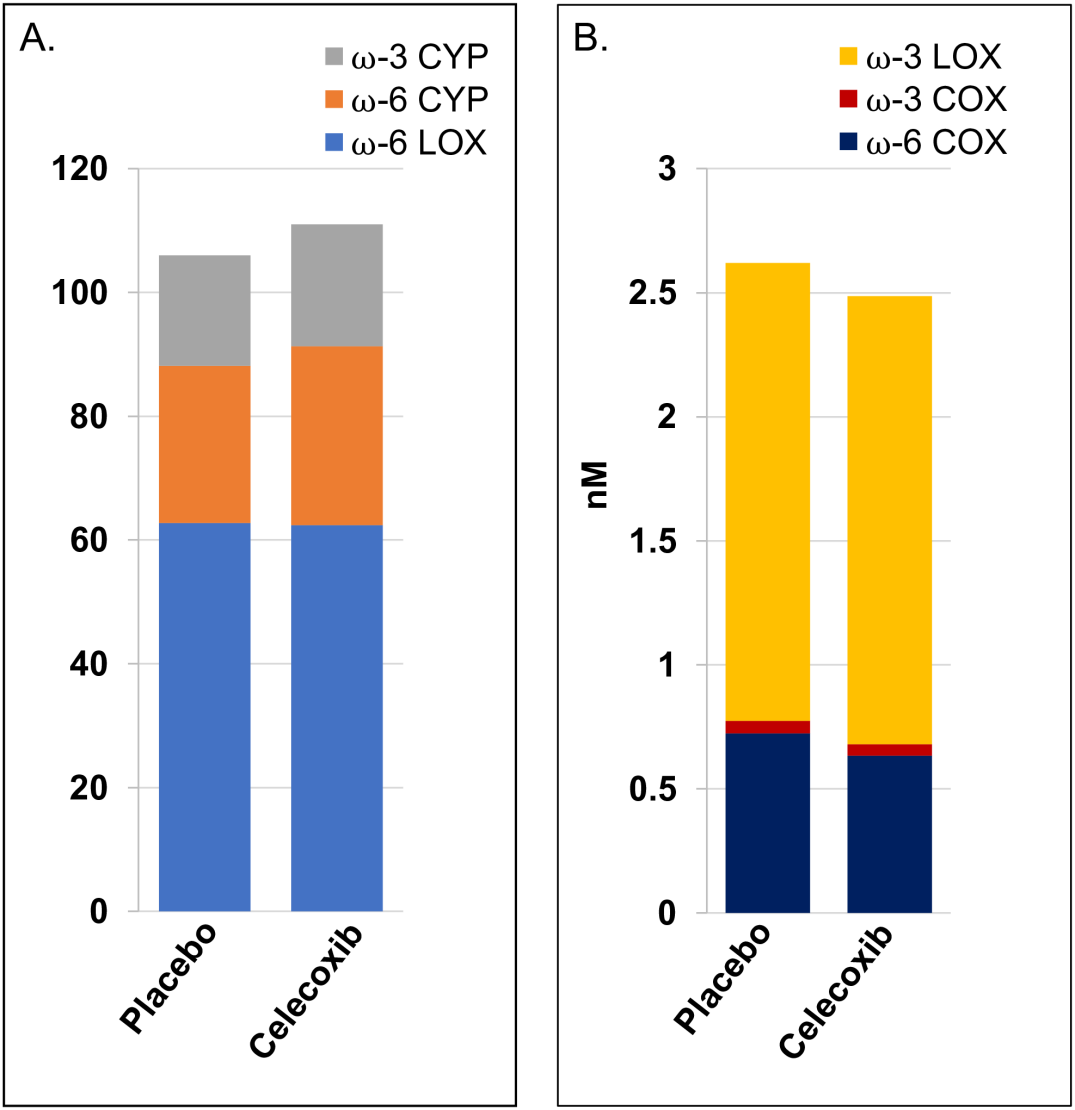
Pathway-based differences for all participants: Celecoxib (*n* = 90) versus placebo (*n* = 95). (A) Summed oxylipins from ω-3 CYP450, ω-6 CYP450, and ω-6 LOX pathway arms. Asterisk (*) indicates *P* = 0.054. (B) Summed oxylipins from ω-3 LOX, ω-3 COX, and ω-6 COX pathways arms. Note that A and B are on different scales. Different colors represent the sum of all quantified oxylipins in a given pathway arm.

After 12 months on study, there were no detectable differences in the summed circulating COX-derived or LOX-derived metabolite concentrations between participants randomized to celecoxib or placebo from either ω-6 or ω-3 fatty acids (Fig 2). Median (IQR) levels of summed CYP450-derived metabolites of ω-6 (but not ω-3) fatty acids were marginally higher in those on celecoxib [28.9 nM (22.2–41.9)] than placebo [25.4 nM (19.4–37.5)] (*P* = 0.054). Individual oxylipin levels between the two groups are shown in Table 2. There were no significant differences in individual COX-derived oxylipins between the celecoxib and placebo arms. In contrast, ARA LOX-derived oxylipin 8-HETE was significantly higher in the celecoxib arm than placebo arm (*P* = 0.005). Additional oxylipins that were significantly higher with celecoxib than placebo were; ω-6 CYP450-derived 5(6)-EpETrE (*P* = 0.045) and 12(13)-EpOME (*P* = 0.023), and ω-3 CYP450-derived 11,12-DiHETE (*P* = 0.042) and 17(18)-EpETE (*P* = 0.047).

**Table 2:** Oxylipin levels, by intervention arm: Median (interquartile range).

Class	PUFA	Pathway	Oxylipin	Placebo (n = 95) nM	Celecoxib (n = 90) nM	Rank-Sum *P*-Value
ω-6	ARA	COX	PGD_2_	0.09 (0.05–0.21)	0.08 (0.05–0.13)	0.096
			PGE_2_	0.16 (0.09–0.28)	0.17 (0.10–0.25)	0.831
			PGF_2α_	0.14 (0.07–0.22)	0.16 (0.07–0.27)	0.293
			TXB_2_	0.06 (0.00–0.21)	0.03 (0.00–0.20)	0.540
			6-keto-PGF_1α_	0.21 (0.03–0.22)	0.06 (0.03–0.22)	0.604
		LOX	5-HETE	1.11 (0.62–1.46)	1.26 (0.77–1.68)	0.063
			5-oxo-ETE	0.57 (0.05–1.49)	0.15 (0.05–1.26)	0.357
			6-trans-LTB_4_	0.37 (0.31–0.47)	0.38 (0.28–0.48)	0.863
			20-OH-LTB_4_	0.04 (0.01–0.14)	0.12 (0.01–0.15)	0.305
			20-COOH-LTB_4_	1.14 (0.61–2.18)	1.29 (0.76–2.44)	0.204
			11,12-,15-TriHETrE	0.24 (0.16–0.40)	0.26 (0.14–0.47)	0.790
			15-HETE	1.45 (1.09–1.83)	1.34 (1.05–2.16)	0.779
			LXA_4_	0.01 (0.01–0.02)	0.01 (0.01–0.02)	0.636
			15-oxo-ETE	0.16 (0.11–0.22)	0.16 (0.12–0.25)	0.548
			12-HETE	0.90 (0.49–1.59)	0.81 (0.43–1.42)	0.503
			12-oxo-ETE	1.88 (0.99–2.96)	1.69 (0.88–2.78)	0.484
			8-HETE^a^	0.00 (0.00–0.00)	0.00 (0.00–0.17)	0.005
			9-HETE	0.80 (0.06–1.23)	0.79 (0.03–1.20)	0.784
			11-HETE	0.09 (0.01–0.31)	0.06 (0.01–0.33)	0.959
		CYP450	20-HETE	0.64 (0.45–0.89)	0.75 (0.54–1.07)	0.094
			5(6)-EpETrE	0.97 (0.67–1.62)	1.19 (0.80–2.10)	0.045
			8(9)-EpETrE	0.34 (0.23–0.58)	0.43 (0.29–0.74)	0.061
			11(12)-EpETrE	0.41 (0.25–0.91)	0.50 (0.32–0.97)	0.128
			14(15)-EpETrE	0.55 (0.38–1.09)	0.70 (0.43–1.20)	0.211
			5,6-DiHETrE	0.28 (0.21–0.38)	0.27 (0.21–0.37)	0.949
			8,9-DiHETrE	0.31 (0.26–0.39)	0.34 (0.27–0.44)	0.258
			11,12-DiHETrE	0.44 (0.35–0.57)	0.44 (0.35–0.61)	0.942
			14,15-DiHETrE	1.05 (0.91–1.38)	1.04 (0.86–1.35)	0.652
			THF diol	0.00 (0.00–0.02)	0.01 (0.00–0.03)	0.186
	LA	LOX	9,10,13-TriHOME	5.10 (2.30–7.86)	4.04 (2.42–9.86)	0.947
			9,12,13-TriHOME	17.3 (12.78–23.4)	18.84 (13.42–26.6)	0.372
			9-HODE	6.94 (4.80–9.38)	6.42 (5.00–8.22)	0.468
			9-oxo-ODE	1.91 (1.20–3.32)	2.51 (1.47–3.58)	0.156
			13-HODE	15.46 (11.92–22.2)	15.11 (11.98–19.22)	0.711
			13-oxo-ODE	0.69 (0.50–0.98)	0.61 (0.43–0.89)	0.171
		CYP450	9(10)-EpOME	4.16 (2.76–6.94)	5.34 (3.36–9.84)	0.061
			12(13)-EpOME	4.56 (3.28–7.80)	5.72 (3.9–10.32)	0.023
			9,10-DiHOME	2.74 (2.12–3.92)	3.09 (2.38–3.98)	0.279
			12,13-DiHOME	6.40 (5.20–9.02)	7.34 (5.36–9.40)	0.210
		Auto-oxidation	EKODE	2.42 (1.35–5.34)	3.17 (1.38–5.80)	0.375
	DGLA	LOX	15(S)-HETrE	0.45 (0.36–0.64)	0.49 (0.35–0.63)	0.794
ω-3	ALA	LOX	9-HOTrE	0.33 (0.25–0.46)	0.33 (0.24–0.42)	0.776
			13-HOTrE	0.41 (0.30–0.59)	0.37 (0.26–0.53)	0.196
		CYP450	9(10)-EpODE	0.39 (0.19–0.76)	0.50 (0.25–1.05)	0.109
			12(13)-EpODE	0.15 (0.07–0.30)	0.19 (0.10–0.40)	0.143
			15(16)-EpODE	2.14 (1.59–3.86)	2.66 (1.58–4.08)	0.512
			9,10-DiHODE	0.02 (0.00–0.08)	0.03 (0.00–0.08)	0.248
			12,13-DiHODE	0.09 (0.05–0.16)	0.11 (0.04–0.18)	0.354
			15,16-DiHODE	5.70 (3.78–8.24)	5.80 (4.00–7.52)	0.854
	EPA	COX	Resolvin E1	0.05 (0.00–0.12)	0.05 (0.00–0.13)	0.754
		LOX	5-HEPE	0.25 (0.17–0.34)	0.27 (0.19–0.43)	0.107
			12-HEPE	0.18 (0.10–0.29)	0.16 (0.09–0.31)	0.602
			15-HEPE	0.09 (0.04–0.14)	0.09 (0.04–0.17)	0.481
		CYP450	8,9-DiHETE	0.04 (0.03–0.07)	0.05 (0.03–0.10)	0.163
			11,12-DiHETE	0.04 (0.03–0.05)	0.05 (0.03–0.07)	0.042
			14,15-DiHETE	0.08 (0.06–0.11)	0.08 (0.05–0.12)	0.919
			17,18-DiHETE	0.41 (0.31–0.57)	0.39 (0.30–0.63)	0.761
			8,15-DiHETE	0.04 (0.01–0.11)	0.06 (0.02–0.14)	0.061
			8(9)-EpETE	0.06 (0.05–0.09)	0.07 (0.05–0.10)	0.095
			11(12)-EpETE	0.06 (0.03–0.10)	0.06 (0.04–0.11)	0.434
			14(15)-EpETE	0.02 (0.00–0.06)	0.02 (0.00–0.06)	0.376
			17(18)-EpETE	0.09 (0.05–0.17)	0.13 (0.07–0.25)	0.047
	DHA	LOX	17-HDoHE	0.40 (0.25–0.71)	0.45 (0.25–0.69)	0.705
		CYP450	7(8)-EpDPE	2.54 (1.49–5.48)	3.10 (1.78–6.62)	0.241
			10(11)-EpDPE	0.25 (0.15–0.42)	0.30 (0.17–0.60)	0.166
			13(14)-EpDPE	0.13 (0.07–0.23)	0.17 (0.09–0.35)	0.094
			16(17)-EpDPE	0.16 (0.09–0.31)	0.19 (0.10–0.36)	0.320
			19(20)-EpDPE	0.35 (0.21–0.65)	0.43 (0.23–0.81)	0.287
			7,8-DiHDPE	0.09 (0.05–0.13)	0.09 (0.06–0.13)	0.585
			10,11-DiHDPE	0.12 (0.09–0.19)	0.14 (0.10–0.23)	0.191
			13,14-DiHDPE	0.13 (0.10–0.20)	0.13 (0.11–0.21)	0.321
			16,17-DiHDPE	0.24 (0.16–0.36)	0.25 (0.18–0.40)	0.580
			19,20-DiHDPE	1.99 (1.35–2.74)	2.03 (1.34–3.02)	0.778
			4,5-DiHDPE	0.48 (0.35–0.74)	0.54 (0.34–1.02)	0.259

^a^The median value for 8-HETE is below the limit of quantitation (< 0.0008 nM).

### Effect of COX-2 inhibition on COX-, LOX-, and CYP450-derived metabolites when stratified by aspirin use

**Fig 3:**
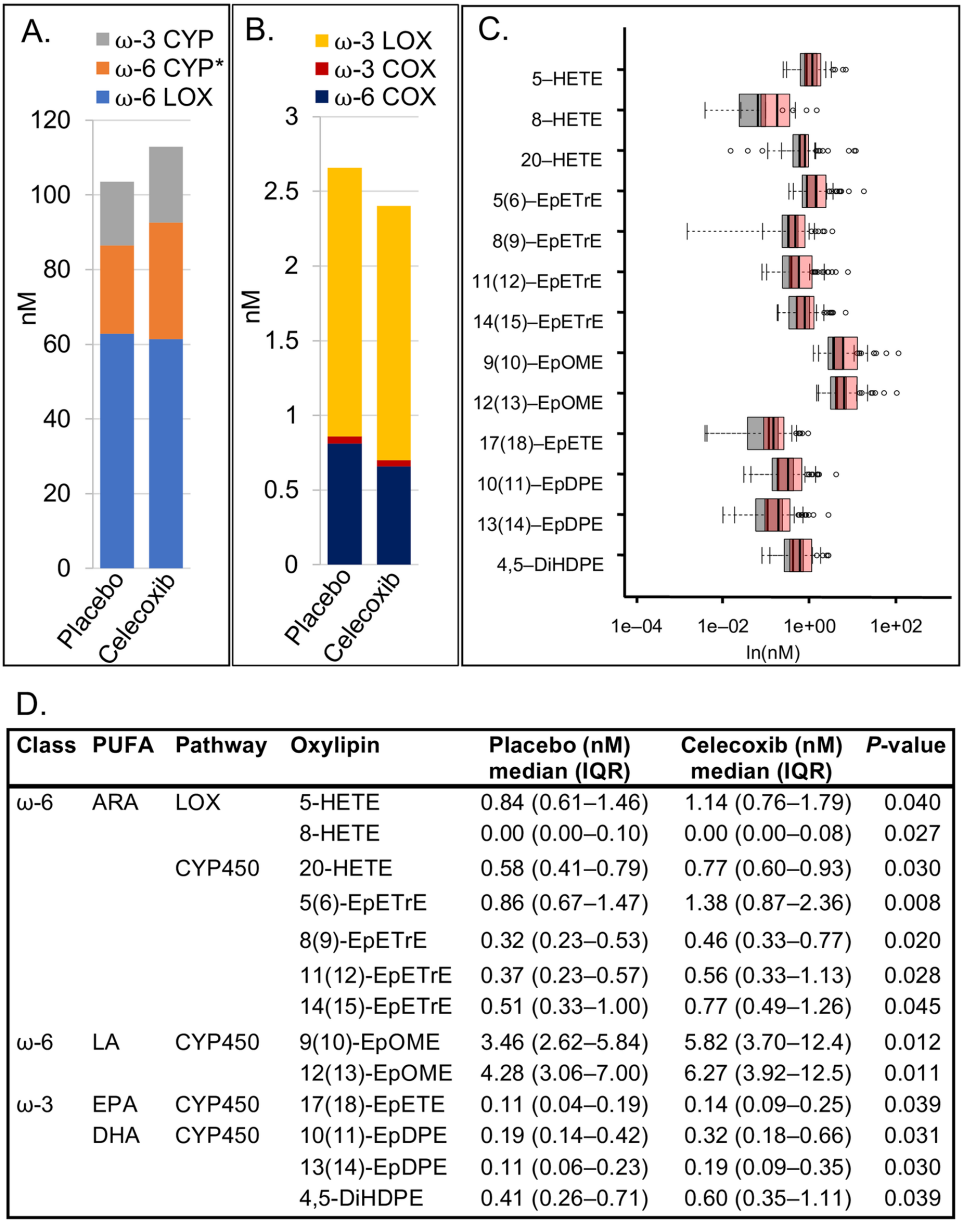
Celecoxib (*n* = 54) versus placebo (*n* = 49) among aspirin non-users only. (A) Summed oxylipins from ω-3 CYP450, ω-6 CYP450, and ω-6 LOX pathway arms. Asterisk (*) indicates *P* = 0.032. (B) Summed oxylipins from ω-3 LOX, ω-3 COX, and ω-6 COX pathways arms. Note that A and B are on different scales. Different colors represent the sum of all quantified oxylipins in a given pathway arm. (C) Overlapping box plots showing significantly different individual oxylipins between placebo (gray) and celecoxib (pink) groups. (D) Median (IQR) levels of oxylipins that are significantly different between groups, with Wilcoxon rank-sum test *P*-values. Note that the median value for 8-HETE is below the limit of quantitation (< 0.0008 nM).

To account for the high prevalent use of low-dose (81 mg/day) aspirin, a non-selective inhibitor of COX-1/2, analyses were stratified by aspirin use. Among aspirin *non-users*, the sum of CYP450-derived oxylipins from ω-6 (but not ω-3) fatty acids was higher in circulation in participants randomized to the celecoxib arm [31.2 nM (22.2–45.1)] than the placebo arm [23.6 nM (19.1–37.5); *P* = 0.032; Fig 3a]. At the individual-oxylipin level, 11 CYP450-derived oxylipins were higher in celecoxib than placebo at *P*<0.05 (Fig 3c and 3d): ARA-derived 20-HETE (*P* = 0.030); 5(6)-EpETrE (*P* = 0.008); 8(9)-EpETrE (*P* = 0.020); 11(12)-EpETrE (*P* = 0.028); and 14(15)-EpETrE (*P* = 0.045); LA-derived 9(10)-EpOME (*P* = 0.012) and 12(13)-EpOME (*P* = 0.011); EPA derived 17(18)-EpETE (*P* = 0.039); and DHA derived 10(11)-EpDPE (*P* = 0.031), 13(14)-EpDPE (*P* = 0.030) and 4,5-DiHDPE (*P* = 0.039). While there were no differences overall in the summed LOX pathway metabolites, 5-HETE and 8-HETE (both ARA-derived) were higher in celecoxib than in placebo (*P* = 0.040 and *P* = 0.027 respectively).

**Fig 4:**
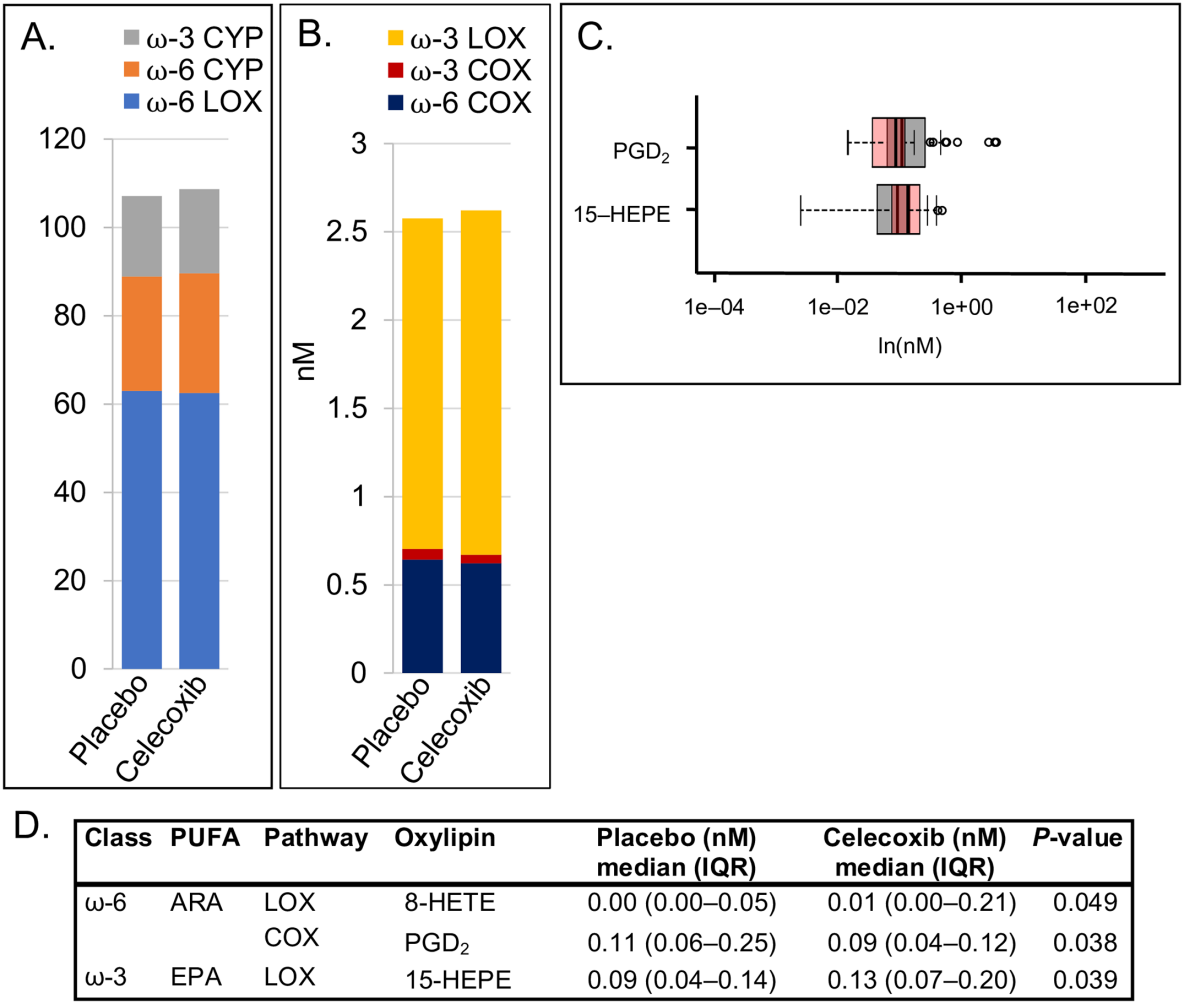
Celecoxib (*n* = 36) versus placebo (*n* = 46) among aspirin users only. (A) Summed oxylipins from ω-3 CYP450, ω-6 CYP450, and ω-6 LOX pathway arms. (B) Summed oxylipins from ω-3 LOX, ω-3 COX, and ω-6 COX pathways arms. Note that A and B are on different scales. Different colors represent the sum of all quantified oxylipins in a given pathway arm. (C) Overlapping box plots showing significantly different individual oxylipins between placebo (gray) and celecoxib (pink) groups. (D) Median (IQR) levels of oxylipins that are significantly different between groups, with Wilcoxon rank-sum test *P*-values. Note that the median value for 8-HETE among the Placebo is below the limit of quantitation (< 0.0008 nM).

Providing evidence of a modulating effect of aspirin, there were no differences at the pathway-level between celecoxib and placebo among aspirin users (Fig 4a). Individually, the ARA metabolite PGD_2_ (a COX product) was higher in the placebo-plus-aspirin group than the celecoxib-plus-aspirin group (*P* = 0.038; Fig 4c and 4d). Additionally, LOX metabolites 15-HEPE (EPA) and 8-HETE (ARA) were higher in celecoxib-plus-aspirin than placebo-plus-aspirin (*P* = 0.039 and *P* = 0.049 respectively).

### Effect of aspirin and any NSAID on COX-, LOX-, and CYP450-derived metabolites

**Fig 5:**
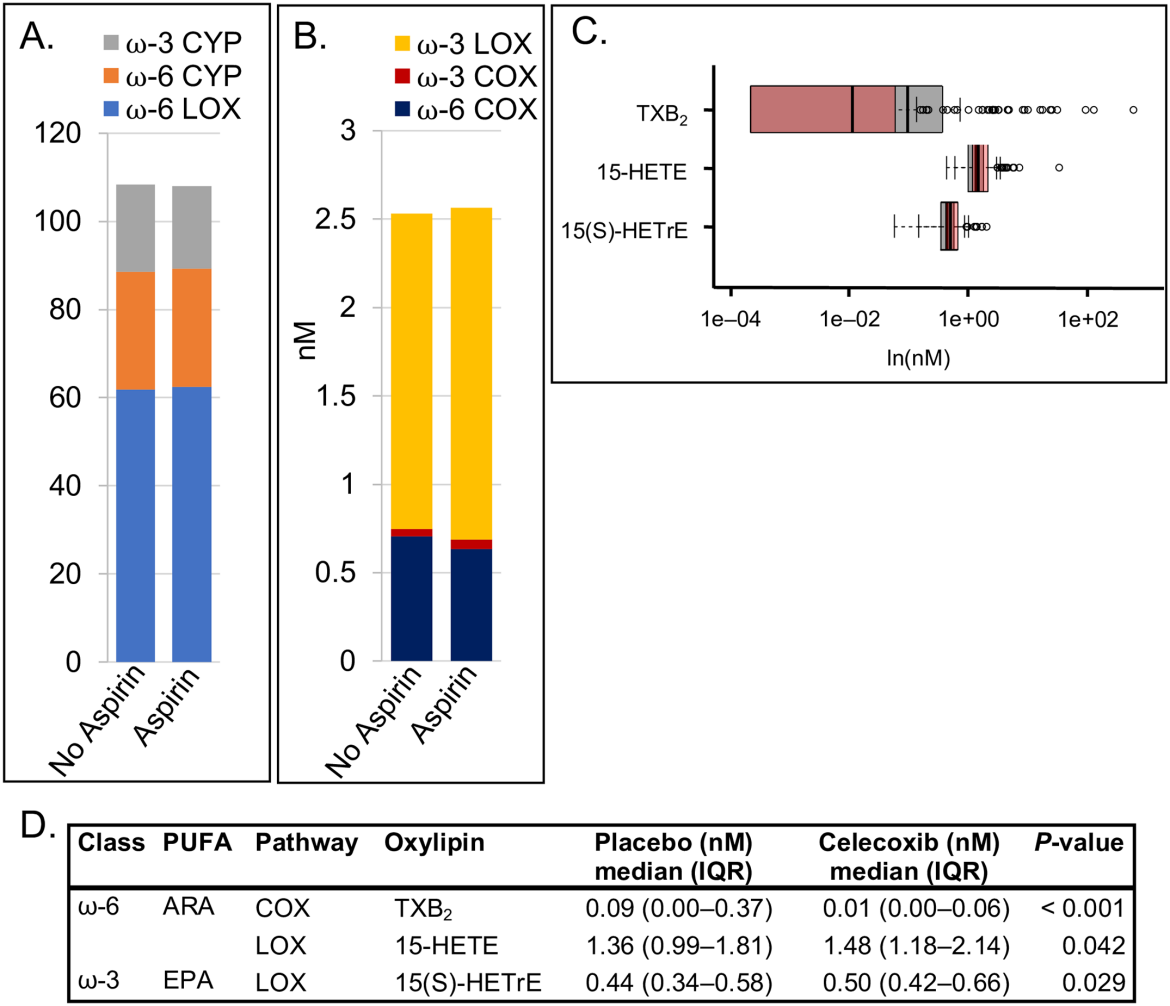
No aspirin (*n* = 103) versus aspirin (*n* = 82) among all participants. (A) Summed oxylipins from ω-3 CYP450, ω-6 CYP450, and ω-6 LOX pathway arms. (B) Summed oxylipins from ω-3 LOX, ω-3 COX, and ω-6 COX pathways arms. Note that A and B are on different scales. Different colors represent the sum of all quantified oxylipins in a given pathway arm. (C) Overlapping box plots showing significantly different individual oxylipins between aspirin non-users (gray) and users (pink). (D) Median (IQR) levels of oxylipins that are significantly different between groups, with Wilcoxon rank-sum test *P*-values.

Next, we considered differences between aspirin users and aspirin non-users with both trial arms combined, to explore aspirin-specific effects. Oxylipins derived from COX metabolism of ω-6 (but not ω-3) fatty acids were non-significantly lower in aspirin users [0.64 (0.43–1.04) nM] compared to non-users [0.71 (0.48–1.47) nM; *P* = 0.109] (Fig 5b). TBX_2_, consistent with being a primary target for aspirin action, was significantly lower (*P* = 0.0003), whereas LOX-derived oxylipins, 15-HETE and 15(S)-HETrE, were significantly higher (*P* = 0.042 and *P* = 0.029, respectively), among aspirin users than non-aspirin users (Fig 5c and 5d). When restricted to *only* the placebo arm to control for effects of celecoxib, only TBX_2_ remained significantly lower in aspirin users compared to nonusers (*P* = 0.012).

**Fig 6:**
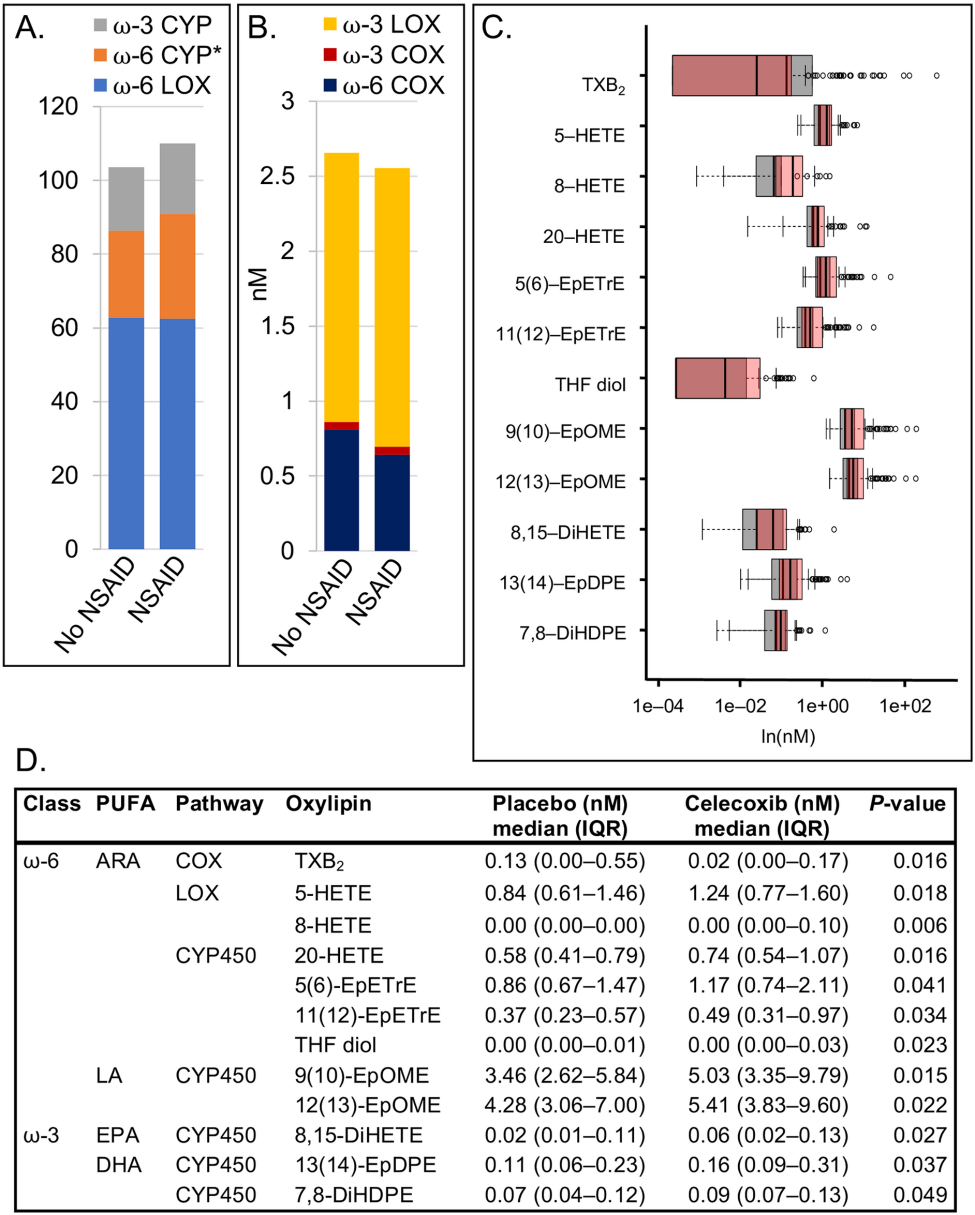
No NSAID (*n* = 49) versus any NSAID (aspirin and/or celecoxib; *n* = 136) among all participants. (A) Summed oxylipins from ω-3 CYP450, ω-6 CYP450, and ω-6 LOX pathway arms. Asterisk (*) indicates *P* = 0.036. (B) Summed oxylipins from ω-3 LOX, ω-3 COX, and ω-6 COX pathways arms. Note that A and B are on different scales. Different colors represent the sum of all quantified oxylipins in a given pathway arm. (C) Overlapping box plots showing significantly different individual oxylipins between NSAID non-users (gray) and users (pink). (D) Median (IQR) levels of oxylipins that are significantly different between groups, with Wilcoxon rank-sum test *P*-values. Note that the median value for 8-HETE is below the limit of quantitation (< 0.0008 nM).

We also considered use of *any* NSAID (i.e., aspirin, celecoxib, or both) in the study sample. Overall, we observed a pattern similar to that of celecoxib alone, with higher CYP450 oxylipin levels derived from ω-6 metabolism in circulation of NSAID users than non-users [28.4 (21.4–40.0) versus 23.6 (19.1–37.5) nM; *P* = 0.036; Fig 6a], with no differences in ω-3 metabolism. Of 12 individual oxylipins that were significantly different between NSAID users and non-users, 11 were higher among NSAID users; 9 of these were CYP450-derived oxylipins that included 20-HETE from ARA (*P* = 0.016; Fig 6c and 6d). In addition, LOX-derived 5-HETE (*P* = 0.018) and 8-HETE (*P* = 0.006) from ARA were also higher in NSAID users. For COX-derived oxylipins, only TBX_2_ from ARA was significantly lower among NSAID users compared to non-NSAID users (*P* = 0.016).

### Oxylipins and blood pressure in the Sel/Cel Trial

We recently reported increased risk of hypertension with daily 400 mg celecoxib in the parent Sel/Cel Trial, observing that the effect of celecoxib on blood pressure was strongest in men [16]. Based on our previous observation that rofecoxib, a potent COX-2 inhibitor in the same class of drug as celecoxib, increases pro-hypertensive 20-HETE in male mice [14] and evidence here for effects of celecoxib on circulating 20-HETE, we next tested for an association between 20-HETE and SBP. In multivariable adjusted analyses, we found no main effect of 20-HETE on SBP (Table 3). However, stratified by sex, we found evidence for a positive association between 20-HETE and SBP in men but not women (*P*_interaction_ = 0.058). In men, a 1-nM increase in 20-HETE was associated with a 2.4-mmHg increase in SBP (*P* = 0.040).

**Table 3:** Associations^1^ between oxylipins and systolic blood pressure overall and stratified by sex^2^.

Class	PUFA	Pathway	Oxylipin	All (n = 185)			Men (n = 126)			Women (n = 59)		
				Beta	SE	*P*-value	Beta	SE	*P*-value	Beta	SE	*P*-value
ω-6	ARA	LOX	LXA_4_	89.6	40.8	0.029	109.5	46.3	0.020	-7.3	93.3	0.938
	ARA	LOX	15-oxo-ETE	17.5	7.4	0.018	23.0	7.7	0.004	-34.8	25.6	0.180
	ARA	LOX	5-HETE	3.5	1.4	0.011	6.0	1.9	0.003	1.1	2.0	0.596
	ARA	LOX	8-HETE	14.7	6.2	0.019	20.5	7.4	0.006	-0.5	11.9	0.966
	ARA	LOX	11,12-,15-TriHETrE	6.0	2.4	0.015	7.1	2.6	0.008	-3.4	7.3	0.644
	ARA	CYP450	20-HETE	1.0	0.9	0.293	2.4	1.2	0.040	-1.1	1.5	0.476
	LA	LOX	13-HODE	0.2	0.1	0.139	0.4	0.2	0.031	-0.1	0.2	0.593
	LA	LOX	9-HODE	0.5	0.3	0.044	0.7	0.3	0.035	0.2	0.5	0.741
ω-3	ALA	LOX	9-HOTrE	17.9	7.1	0.013	27.1	9.2	0.004	3.8	11.4	0.743
	EPA	LOX	12-HEPE	3.9	1.6	0.016	3.9	1.7	0.023	2.9	6.6	0.661
	DGLA	LOX	15(S)-HETrE	9.5	4.2	0.024	15.3	5.1	0.003	-3.3	8.2	0.692

^1^Adjusted for age, sex, BMI, aspirin, hypertension medication, and celecoxib

^2^Only oxylipins that were significantly correlated with systolic blood pressure in at least one analysis are shown

Because 20-HETE is one of many oxylipins implicated in vascular tone, we also explored the associations between all measured oxylipins and SBP. In analyses of men and women combined, 9 oxylipins, all derived from LOX mediated metabolism, were found to be positively associated with SBP (Table 3). Of these, the LOX-derived LXA_4_, 15-oxo-ETE, 8-HETE, and 9-HOTrE exhibited the greatest effect on SBP, consistent with published studies demonstrating an important role of LOX enzyme in hypertension [21]. Analyses stratified by sex suggest that associations between oxylipins and SBP are stronger in men than women. However, the test for oxylipin-by-sex interaction on SBP was *P*<0.1 for only LOX-derived 13-HODE, 15(S)-HETrE, and 15-oxo-ETE and CYP450-derived 20-HETE (*P* = 0.064, *P* = 0.064, *P* = 0.055, and *P* = 0.058 respectively).

## Discussion

Utilizing a mass spectrometry approach to profile 74 oxylipins from COX, LOX, and CYP450 metabolism of PUFAs, we found no evidence for differences in plasma COX-derived metabolites between participants randomized to a 400-mg daily dose of celecoxib or its placebo after 12 months on intervention. In contrast, plasma levels of 4 CYP450-derived metabolites and LOX-derived 8-HETE, were higher in the celecoxib arm compared to placebo. Among non-aspirin users there were 11 CYP450-derived metabolites and 2 LOX-derived. Of these, 2 ARA-derived oxylipins (e.g., LOX-derived 8-HETE and 5-HETE) were positively associated with blood pressure overall with a stronger association among men, and CYP450-derived 20-HETE was positively associated with blood pressure among men only.

In the initial hypothesis testing, it was expected that levels of COX-derived metabolites, in particular PGE_2_, would be lower in the celecoxib group relative to placebo. However, only 7 COX-derived oxylipins exhibited sufficient detectability in plasma and, of those, 4 had > 60% of measurements below the limit of quantitation. This finding is consistent with the instability of these metabolites in circulation and highlights the challenges associated with quantifying the unstable products of the COX-2 pathway [22]. Thus, interpretation of celecoxib effects on the COX pathway metabolites should be interpreted with caution.

CYP450 metabolites derived from ω-6 fatty acids, including 20-HETE, are strongly suspect as causal in hypertension and cardiotoxicity of the ω-6 fatty acids [23]. In this study, the only CYP450 ω-6 derived metabolites that were significantly higher in the celecoxib than placebo arms were two putative cardioprotective epoxygenase metabolites: 5(6)-EpETrE and 12(13)-EpOME, derived from ARA and LA, respectively [24]. In addition, two ω-3 CYP450 oxylipins, 11,12-DiHETE and 17(18)-EpETE, both derived from EPA, were found to be present at higher plasma concentrations in the celecoxib group. These epoxygenase metabolites from ω-6 and ω-3 are metabolized by the soluble epoxide hydrolase (sEH) enzyme to corresponding 1,2-dihydroxy-fatty acids (i.e., DiHOMEs and DiETEs); which have been positively associated with cardiotoxic effects [25]. Preclinical studies have shown that inhibition of the sEH enzyme leading to increases in the EpOME/DiHOME and EpETE/DiHETE ratios has cardioprotective activity [26, 27]. In our study, we found no differences between placebo and celecoxib groups in either of these ratios or stratified by sex (*data not shown*) suggesting no effect of celecoxib on sEH. Additional studies however are needed to confirm the lack of celecoxib effects on cardiac outcomes due to perturbing of the epoxygenase metabolites.

Some CYP450 oxylipins have potent microcirculatory effects, with adverse effects on the vasculature as well as renal, cerebral, and cardiac health [25]. 20-HETE, a CYP450-derived ω-hydroxylation metabolite of ARA, is among the better-characterized vascular-acting oxylipins [28]. 20-HETE plays a complex role in blood pressure regulation, including a proposed prohypertensive activity resulting from induction of the endothelial angiotensin-converting enzyme and stimulation of the renin–angiotensin–aldosterone system. Previously, we found that administration of the COX-2-selective inhibitor rofecoxib resulted in a 120-fold increase in 20-HETE in the plasma of male mice and that administered 20-HETE shortened tail bleeding time (a bioindicator for cardiotoxic activity) [13]. Plasma 20-HETE levels in this study were found to be non-significantly increased in the celecoxib arm compared to placebo, and significantly higher in participants on celecoxib not taking aspirin when compared to the placebo arm not taking aspirin (Fig 3c and 3d). There was no evidence for similar dramatic increases in 20-HETE levels with celecoxib in either men or women as observed with rofecoxib in male mice. This suggests that the observed increase in 20-HETE levels in mice is either specific to rofecoxib, shown to have higher cardiovascular toxicities, and/or that COX-2 inhibition and effects on 20-HETE metabolism differ between mice and humans. Our observation of a positive association between 20-HETE and blood pressure in men however does support a role for 20-HETE on vascular tone. The lack of an association in women is interesting given evidence that 20-HETE mediates both androgen-induced and androgen-independent hypertension in experimental models [29]. The contribution of sexual dimorphism in NSAID-specific adverse events, including a possible role for androgen-regulated CYPs in ARA metabolism [30], is intriguing and merits further investigation.

In addition to the effects of COX-2 inhibition on CYP450 metabolism of PUFAs, LOX metabolism has been reported to be disturbed with inhibition of COX-2 and is supported by our findings. Two LOX-derived oxylipins from ARA, 5-HETE and 8-HETE, were higher in celecoxib compared to placebo. Plasma concentrations of 8-HETE were increased with celecoxib, independent of aspirin, and positively associated with SBP. Notably, 8-HETE was the only metabolite of ARA that was significantly higher in individuals on both celecoxib and low dose aspirin. We did not observe independent effects of aspirin on 8-HETE, though our sample size for this group is limited. Further work is needed to determine if this represents any additive effects of the two drugs. While less characterized for its effects, 8-HETE has been shown to be pro-inflammatory and to induce cardiac hypertrophy in cultured cardiomyocytes [31]. Overall, 5-HETE was higher in the celecoxib arm compared to placebo (but not statistically significant), and among non-aspirin users was statistically significantly higher in the celecoxib arm. Similar to 8-HETE, 5-HETE was also positively associated with SBP. The association with SBP is supported by pre-clinical studies and results from clinical trials where 5-HETE has been shown to induce pulmonary vasoconstriction as well as play a role in the development of hypertension and the pathogenesis of cardiac hypertrophy [32].

When any NSAID (aspirin, celecoxib, or both) was compared to no NSAID use (Fig 6), we observed differences between the groups in the level of plasma CYP450 metabolites derived from ω-6 fatty acids with a pattern similar to celecoxib alone. There were 9 total CYP450-derived metabolites that were significantly higher in NSAID users, including 20-HETE. As with celecoxib alone, increases in the putative cardioprotective epoxygenase metabolites 5(6)-EpETrE (from ARA), 11(12)-EpETrE (from ARA), and 13(14)-EpDPE (from DHA) were significantly higher among NSAID users [25]. However, among the any NSAID use group, sEH-derived metabolites 8,15-DiHETE (from EPA) and 7,8-DiHDPE (from DHA), were significantly higher. In addition to these metabolites, two LA oxylipins from CYP450 metabolism (i.e., 9(10)-EpOME and 12(13)-EpOME) were significantly higher among NSAID users. Their significance is unclear. The literature on LA oxylipins is limited and inconsistent; some, but not all, preclinical studies suggest these oxylipins are cardiotoxic [33]. Analysis of the EpOME/DiHOME and EpETE/DiHETE ratio for any NSAID use versus no NSAID use in all subjects or sex-stratified analyses were null (data not shown).

To gain further insight on the relationship between circulating oxylipins and blood pressure independent of NSAIDs, we also investigated the association between the individual oxylipins and SBP controlling for NSAIDs, age, sex and medication for hypertension and stratified on sex. Three of the oxylipins that exhibited differences between the celecoxib and placebo arms (5-HETE, 8-HETE and 20-HETE) were significantly different when the analysis was restricted to non-aspirin users and also were independently associated with SBP. This strengthens the potential significance of COX-2 inhibitor perturbations on these metabolites in risk for hypertension. It is noteworthy that 9 of the 11 oxylipins found to be independently and positively associated with SBP were derived from LOX mediated metabolism. Of these, LXA_4_, 15-oxo-ETE, 8-HETE and 9-HOTrE exhibited the greatest effect on SBP. The predominant involvement of the LOX derived metabolites is interesting given limited, but compelling, experimental evidence demonstrating an important role of LOX enzymes in experimental hypertension in mice [21]. Interpreting these results and their significance for hypertension is challenging given the unclear relationship between mouse and human LOX enzyme/metabolite systems and physiologic effects. As an aside, for all the oxylipin associations with SBP, the associations were more striking for men and in some cases test for interaction were suggestive of sex specific differences. However, these should be interpreted with caution and considered only hypothesis generating due in part to smaller numbers of women in the study and the influence of other uncontrolled factors including dietary differences in fatty acids between men and women or actual adherence to blood pressure medications.

## Conclusions

Here we provide evidence from a randomized trial that celecoxib use increases specific circulating CYP450- and LOX-derived oxylipin metabolites that are positively associated with measured blood pressure. Overall effects of celecoxib use on CYP450 and LOX derived metabolites were more evident in participants who did not report taking regular aspirin on study. This result suggests modulating effects of aspirin in combination with COX-2 inhibitors on circulating oxylipins. Importantly, this is the first study to identify LOX ARA derived 8- and 5-HETE as putative mediators of celecoxib adverse effects on blood pressure. While our findings require replication, and are hypothesis-generating in nature, this is the first report on celecoxib effects on plasma oxylipin levels using a large mass spectrometry panel of oxylipin metabolites in a randomized sample. Further, it is the first to support effects of COX-2 inhibitors on 20-HETE and relate this to blood pressure in men.

## Supporting information

S1 Fig Metabolism of ω-6 and ω-3 fatty acids. ω-6 (panel A) and ω-3 (panel B) fatty acids are metabolized by three enzyme families, cyclooxygenase (COX), lipoxygenase (LOX), and cytochrome P450 (CYP450), to produce multiple bioactive metabolites called oxylipins. CYP450 metabolites are metabolized by soluble epoxide hydrolase (sEH). Only oxylipins that were quantified on our analytical platform are depicted, excluding THF diol which is produced from multiple epoxyeicosatrienoic acids. Enzymes are shown in grey boxes.
(PDF)
