# Supplementary figures and images for: Celecoxib use and circulating oxylipins in a colon polyp prevention trial
Source: PLoS One. 2018 Apr 26;13(4):e0196398. doi: 10.1371/journal.pone.0196398 (PMC5919576; doi:10.1371/journal.pone.0196398)

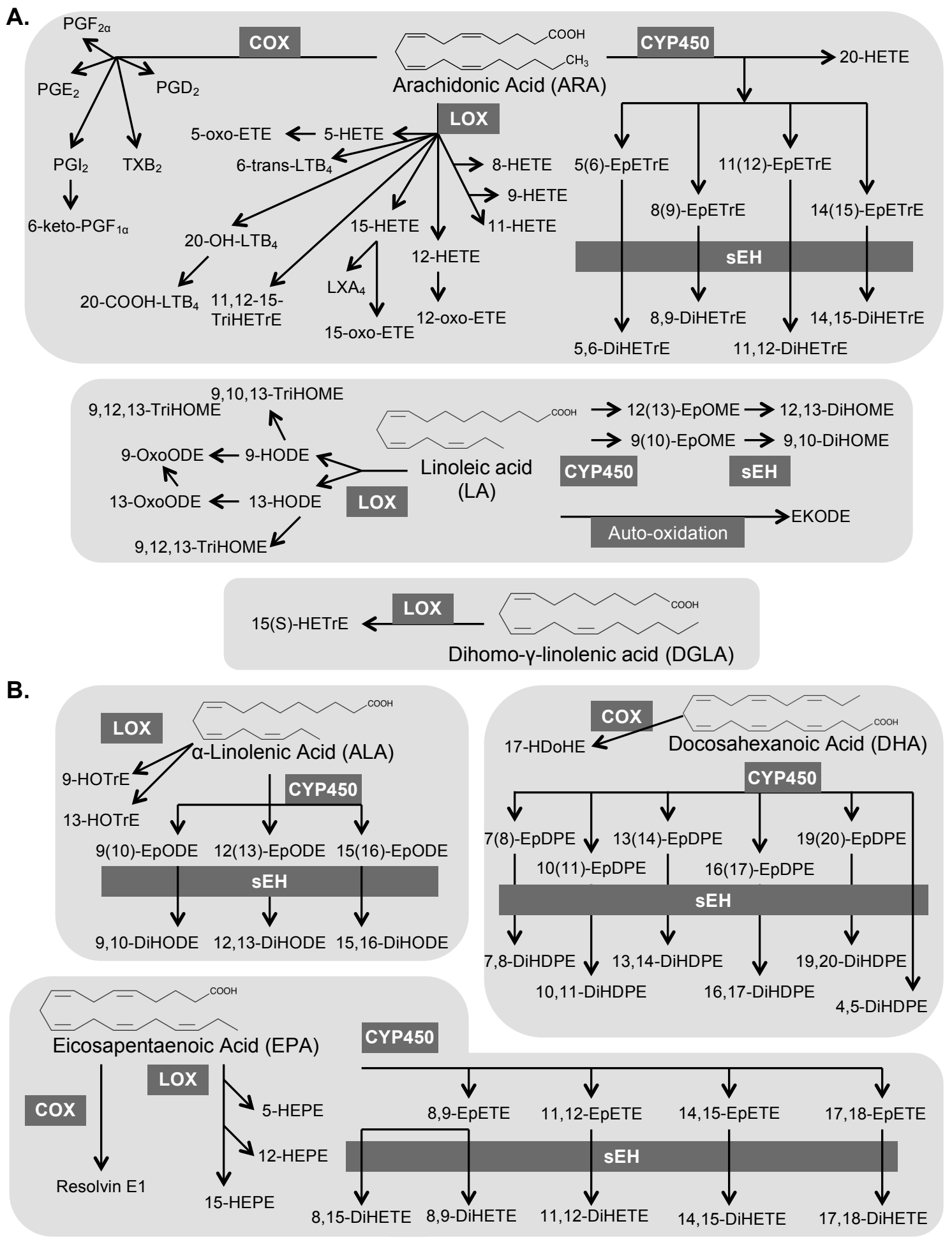

Supplement: S1 Fig — ω-6 (panel A) and ω-3 (panel B) fatty acids are metabolized by three enzyme families, cyclooxygenase (COX), lipoxygenase (LOX), and cytochrome P450 (CYP450), to produce multiple bioactive metabolites called oxylipins. CYP450 metabolites are metabolized by soluble epoxide hydrolase (sEH). Only oxylipins that were quantified on our analytical platform are depicted, excluding THF diol which is produced from multiple epoxyeicosatrienoic acids. Enzymes are shown in grey boxes. (PDF) [file pone.0196398.s001.pdf]
